# Prevalence and risk factors of dry eye symptoms in Jazan Province, Saudi Arabia: a cross-sectional study

**DOI:** 10.3389/fopht.2025.1610763

**Published:** 2025-10-31

**Authors:** Hatim Hassan Najmi, Abdulrahman Mohsen Tubayqi, Sultan Mousa Bakri, Abdullah Meshal Alsharif

**Affiliations:** 1 Oculoplastic Division, Department of Ophthalmology, Dhahran Eye Specialist Hospital, Dhahran, Saudi Arabia; 2 Department of Emergency, Prince Mohammed Bin Nasser Hospital, Jazan, Saudi Arabia; 3 Cornea and External Eye Disease Division, King Saud University, Riyadh, Saudi Arabia; 4 College of Medicine, Umm AlQura University, Makkah, Saudi Arabia

**Keywords:** dry eye, prevalence, Saudi Arabia, ocular surface disease index, risk factors

## Abstract

**Purpose:**

Dry eye syndrome (DES) is characterized by tear film and ocular surface disruptions. Symptomatic DES, one of the most common ocular diseases, reduces the quality of life. This study aimed to evaluate the prevalence of and possible risk factors for DES in the Jazan region of Saudi Arabia.

**Methods:**

This observational, cross-sectional study was conducted between October 2018 and May 2023. This study was conducted with 1061 participants using an online survey that included questions on sociodemographic characteristics, dry eye symptoms, possible factors related to dry eye, and chronic comorbidities. Dry eye symptoms were evaluated using the Ocular Surface Disease Index (OSDI).

**Results:**

The overall prevalence of dry eye symptoms was 59.9%, with 19.7% of the respondents having mild, 14.0% having moderate, and 26.2% having severe dry eye symptoms. A statistically significant difference in dry eye symptoms prevalence was observed between males and females (X^2^ = 54.167; p = 0.000), with females (68.4%) being more commonly affected than males (52.2%). Moreover, Female participants were 1.78 times more likely to develop DES than males, and this association remained statistically significant after adjustment (OR = 1.78; 95% CI: 1.37–2.31). Smoking (p = 0.44), computer use (p = 0.87), and mobile phone use (p = 0.69), were not significantly associated with DES prevalence.

**Conclusion:**

Dry eye symptoms are highly prevalent in Jazan Province, Saudi Arabia, affecting nearly 60% of the population. Female sex was a significant risk factor, while screen time and smoking were not. However, as the study relied on self-reported symptoms without objective clinical tests, the findings may not fully reflect disease prevalence. These results highlight the need for targeted screening and public health efforts in high-risk groups.

## Introduction

Dry eye syndrome (DES) is characterized by tear film and ocular surface disruptions caused by multiple factors (1–3). It presents as eye discomfort, visual disturbances, and tear film instability. Symptoms can range from mild irritation to severe burning, ocular fatigue, and pain, ultimately affecting daily functioning (4–6). Although it rarely causes vision loss, symptomatic DES inevitably reduces the quality of life and represents one of the most common ocular complaints, leading to substantial economic burden with an estimated 7–10 million Americans requiring artificial tears and an annual cost of over $100 million (4, 7–10).

DES prevalence varies by whether diagnosis is symptom or sign based so standardized methods are needed for consistency (11–14). This variation can be attributed to the different case definitions used, populations surveyed, and methodologies used (4, 7, 15, 16). For example, DES prevalence was 15.3% in the Blue Mountain Study (17), 14.5% in the Beaver Dam Study (18), and 33.7% in the Shiphai Eye Study (4). Studies involving tear function tests to determine dry eye have generally reported lower DES rates. The tests used in these studies included Schirmer’s test, fluorescein staining, tear break-up time, and rose bengal staining (19, 20). Only a few studies have reported the subtype-based prevalence of DES. The most common subtype in these studies was lipid anomaly, followed by aqueous tear deficiency and mucin layer deficiency (21, 22).

There are numerous environmental and epidemiological risk factors that have been identified for dry eye, including female gender (16, 24, 26, 27), advanced age (18, 20, 23), systemic conditions such as diabetes or thyroid disease, smoking (24), contact lens wear (25), and pterygium (4). In addition, climatic conditions contribute to dry eye; research from Saudi Arabia and other locations demonstrated that hot and arid weather with high reliance on air conditioning are presenting risk factors for dry eye (7, 26, 27). Furthermore, although prevalence has been documented from regions such as Alahsa (32.1%) (7), numbers from Jazan Province have been less well-documented. Taking into account Jazan’s environment with the potential of prolonged exposure to high temperatures, dust, and a combined urban and rural population, further investigation is warranted in Jazan Province. The aim of the current study was to assess the prevalence of dry eye symptoms and risk factors in Jazan Province, Saudi Arabia. To the best of our knowledge, this is the first study to evaluate the prevalence and risk factors of dry eye symptoms in a large population-based sample in the Jazan region and among a small population in Saudi Arabia.

## Methods

### Study design

This observational, cross-sectional study was conducted between October 2018 and May 2023 to evaluate the prevalence and risk factors of dry eye symptoms and identify possible risk factors in Jazan Province, Saudi Arabia. Jazan is located in the southwestern region of Saudi Arabia, bordering the Red Sea and Yemen. The province has a hot desert climate with high temperatures, low humidity, and frequent dust exposure conditions that may influence ocular surface health. Jazan has a population of approximately 1.4 million people, with both urban and rural communities.

### Study population

Each participant completed the survey once. The extended data collection period reflects the phased, voluntary nature of online recruitment across Jazan, driven by limited resource availability.

The inclusion-exclusion criteria of this study were as follows:

#### Inclusion criteria

1. Male and female participants.
2. Saudi and non-Saudi individuals.
3. Aged 18 years or older.
4. Any individual residing in Jazan for the last 6 months.

#### Exclusion criteria

1. Participants below 18 years of age.
2. Any person from Jazan who stayed there for less than 6 months in the last 6 months.

These criteria were established to ensure that the study included a diverse range of participants within the targeted population, while maintaining consistency and relevance to the objectives of evaluating the prevalence and the risk factors of dry eye symptoms and identifying potential risk factors in Jazan Province, Saudi Arabia. participants under 18 were excluded because the Ocular Surface Disease Index [OSDI] questionnaire is validated for adults, and its reliability in younger populations has not been established.

### Sampling

Convenience sampling was employed, as the author recruited participants through an online survey distributed via various social media websites and applications. This method involved selecting individuals who were readily available and accessible, specifically those with access to online surveys via social media platforms. No randomization or stratified sampling procedures were employed unless what is mentioned before.

### Study variables and measurement tool

The third part assessed the prevalence of dry eye symptoms using the OSDI. It consists of 12 questions rated from 0 to 4, with a total score of 100, calculated using the following equation (17, 28):


$$OSDI=\frac{\left(sum\;of\;s\;core\right)\times 25}{number\;of\;questions\;answered}$$

The OSDI scores were then categorized as normal (0–12), mild (13–22), moderate (23–32), and severe (33–100) ocular surface (17). The total OSDI was then divided by 100 to change the score from 0–100 to 0–1 to solve the possible problem of skewed distribution (29).

Finally, the fourth part asked about the potential risk factors for DES. The Arabic survey was pretested on a random sample of 10 participants (who were not included in the final analysis) for understandability and clarity.

Compared to that by other diagnostic scales, the OSDI provides a more comprehensive assessment of DES by considering the severity of symptoms, functional limitations, and their influence on daily activities. This multifaceted approach allows for a more accurate estimation of the overall burden of DES on individuals. Furthermore, the subjective nature of dry eye symptoms was well captured by the OSDI through self-reported responses. By allowing individuals to express the frequency and intensity of their symptoms, the OSDI provides valuable insights into their life experiences. Given the advantages of the OSDI, its use in estimating DES prevalence is crucial for a more holistic understanding of the condition. By incorporating the OSDI into research studies, researchers can obtain a comprehensive assessment of the impact of DES on individuals’ lives, enhancing our understanding of the prevalence and severity of the disease.

### Data collection

An online link to the survey was sent to the participants through various social media websites and applications, such as WhatsApp, through Google Forms. The author translated the survey from English to Arabic using back-translation. The study utilized a previously validated Arabic version of the (OSDI) questionnaire. No new psychometric validation was conducted. The Arabic OSDI has demonstrated robust validity and reliability in prior studies (28) (30),. The online survey comprised four sections. The first part ensured the participants’ anonymity and stated the aims of the study. After providing informed consent at the beginning of the survey (Are you a resident of the Jazan region and agree to participate in this survey)?, the participants were directed to the next part. The second part included demographic information, such as age, sex, residence, and employment.

### Statistical analysis

Data analysis was performed using the Statistical Package for the Social Sciences (SPSS) version 21 (SPSS Inc., Chicago, IL). Qualitative and quantitative variables were measured as frequencies, mean, median, standard deviation (SD). Chi-square tests were used to assess associations between categorical variables and dry eye severity. A t-test was performed to compare different age groups. The level of significance was set at p<0.05. Multivariate logistic regression was conducted (Model fit was evaluated using the Hosmer–Lemeshow goodness-of-fit test) to identify independent predictors of DES. Covariates were selected based on existing literature and theoretical relevance, including sex, age, smoking, screen time, and refractive surgery. Model fit was evaluated using the Hosmer–Lemeshow goodness-of-fit test, and multicollinearity was assessed via Variance Inflation Factors (VIFs). All VIFs were <2, indicating acceptable levels of collinearity. Interaction terms were not included in the model to preserve statistical power and model simplicity. The online survey required mandatory responses for all items, preventing missing data. Accordingly, all analyses were conducted on complete datasets without the need for imputation.

Odds ratios (ORs) were reported with their corresponding 95% confidence intervals (CIs). The width of each CI was considered in interpreting the precision of the estimates; narrow CIs were taken to indicate greater precision and reliability of the association, whereas wide CIs indicated greater uncertainty.

## Results

A total of 1061 participants (552 males and 509 females) participated in this study However, Since the survey was distributed through open social media channels, an exact response rate could not be determined. The average age (± SD) of respondents was 29.5 (± 9.4) years, and the majority (72.5%) were in the 21–40 age group. A vast majority (99.1%) of the respondents were Saudis, and most (73.8%) had a bachelor’s degree or diploma. Married respondents constituted 53.0% of the sample. The majority (63.5%) had a job, and 46.3% had a monthly income of >5000 Saudi Riyals (**Table 1**).

**Table 1:** Sociodemographic characteristics of the study sample (*N* = 1061).

Variables	*n* (%)
Sex		
Male	552 (52.0)
Female	509 (48.0)
Age (in years)		
≤20	157 (14.8)
21–40	769 (72.5)
>40	135 (12.7)
Nationality		
Saudi	1051 (99.1)
None-Saudi	10 (0.9)
Educational level		
Elementary school	9 (0.8)
Middle school	29 (2.7)
Secondary school	194 (18.3)
Bachelor or Diploma degree	783 (73.8)
Post graduate	46 (4.3)
Marital status		
Single	471 (44.4)
Married	562 (53.0)
Divorced/widow	28 (2.6)
Job status		
Student	338 (31.9)
Working	674 (63.5)
Retired	23 (2.2)
Self-employed	26 (2.5)
Monthly income (Saudi Riyals)	
< 5000	491 (46.3)
5000–1000	265 (25.0)
> 10000	305 (28.7)

**Table 2** describes the respondents’ behavioral and health background details. Of the 1061 respondents, 15.6% were smokers, 73.8% used computers >3 h/day, and 49.3% used mobile phones >6 h/day. Regarding health background, 10.7% of the patients had a history of refractive surgery.

**Table 2:** Behavioral and health background characteristics of the study sample (*N* = 1061).

Variables	*n* (%)
Smoking	
Yes	166 (15.6)
No	895 (84.4)
Computer use (in hours)	
< 3	783 (73.8)
3–6	177 (16.7)
>6	101 (9.5)
Mobile phone use (in hours)	
< 3	138 (13.0)
3–6	400 (37.7)
>6	523 (49.3)
Refractive surgery	
Yes	114 (10.7)
No	947 (89.3)
Role of environmental factors in (DES)	
Hot and dry weather	420 (39.6)
Air pollution	285 (26.9)
Dust and sand particles in the air	321 (30.2)
Exposure to air conditioning or heaters	460 (43.3)
Lack of humidity	380 (35.8)
All of the above	150 (14.1)
None of the above	90 (8.5)

^*^ Including many conditions with very low frequencies, such as sebaceous cyst.

As shown in **Table 3**, the overall prevalence of DES was 59.9%, with 19.7%, 14.0%, and 26.2% of the respondents having mild, moderate, and severe DES, respectively. There was a statistically significant difference in DES prevalence between males and females (X^2^ = 54.167; p= 0.000), with females (68.4%) being more commonly affected than were males (52.2%).

**Table 3:** Prevalence of mild, moderate, and severe DES, distributed by sex and age (n=1061).

Variable	Severity of DES (OSDI scores)				*p* value
	Normal (0–12)	Mild (13–22)	Moderate (23–32)	Severe (33–100)	
Sex *n* (%)					
Male	264 (47.8)	123 (22.3)	65 (11.8)	100 (18.1)	0.000
Female	161 (31.6)	86 (16.9)	84 (16.5)	178 (35.0)	
Total *N* (%)	425 (40.1)	209 (19.7)	149 (14.0)	278 (26.2)	1061 (100.0)
Age (in years)					
≤20	58 (36.9)	30 (19.1)	26 (16.6)	43 (27.4)	0.066
21–40	323 (42.0)	154 (20.0)	107 (13.9)	185 (24.1)	
>40	44 (32.6)	25 (18.5)	16 (11.9)	50 (37.0)	
Total *N* (%)	425 (40.1)	209 (19.7)	149 (14.0)	278 (26.2)	1061 (100.0)

DES, dry eye syndrome; OSDI, Ocular Surface Disease Index.

Regarding the age distribution of dry eye symptom prevalence, respondents aged >40 years (67.4%) were the most frequently affected, followed by those aged ≤20 years (63.1%). No statistically significant difference in DES prevalence was observed between the different age groups (X^2^ = 11.812; p= 0.066).

In the bivariate analysis (**Table 4**), female sex (OR = 1.98; 95% CI: 1.54–2.55) and history of refractive surgery (OR = 2.22; 95% CI: 1.42–3.46) were significantly associated with dry eye symptoms. In the multivariate logistic regression, only female sex remained a significant predictor (adjusted OR = 1.78; 95% CI: 1.37–2.31; p < 0.001), while refractive surgery did not retain significance (adjusted OR = 1.53; 95% CI: 0.95–2.47; p = 0.08). The final regression model demonstrated good fit (Hosmer–Lemeshow p = 0.61), and no multi-collinearity was detected (all VIFs < 2).

**Table 4:** Evaluation of risk factors for DES using bivariate and multivariate logistic regression analyses.

Variable	Unadjusted			Adjusted		
	OR	95%CI	p value	OR	95%CI	p value
Sex						
Male	1			1		
Female	1.98	1.54–2.55	0.000	1.78	1.37–2.31	0.000
Age (in years)						
≤20	1					
21–40	0.81	0.57–1.15	0.24			
>40	1.21	0.75–1.97	0.44			
Computer use (in hours)						
< 3	1					
3–6	1.03	0.74–1.44	0.87			
>6	1.03	0.67–1.57	0.90			
Mobile phone use (in hours)						
< 3	1					
3-6	0.79	0.53–1.17	0.23			
>6	0.92	0.63–1.63	0.69			
Refractive surgery						
No	1			1		
Yes	2.22	1.42–3.46	0.000	1.53	0.95–2.47	0.08
Smoking						
No	1					
Yes	1.14	0.82–1.60	0.44			

DES, dry eye syndrome; OR, odds ratio; CI, Confidence interval.

## Discussion

This study found that nearly 60% of participants in Jazan Province reported dry eye symptoms, with female sex emerging as a significant risk factor. In contrast, smoking, screen time, and refractive surgery were not independently associated with dry eye symptoms. Recent systematic reviews and meta-analyses indicate the prevalence of (DES) varies globally. McCann et al. (31) reported general population prevalence rates of 13% in Brazil and 41% in Mexico, with significantly higher rates among specific groups such as indoor workers (70%), students (71%), and in general ophthalmology clinics (83%) (31). Bayesian analysis by Papas (32). estimated the average DES prevalence at 11.59%, with symptomatic cases around 9.12% and notable differences between genders 9.5% in women and 6.8% in men. The lowest prevalence was observed in North America at 4.6%, while Africa reported the highest at 47.9%. Furthermore, a 2024 systematic review by Loaiza-Guevara et al. (33) reported that the prevalence of dry eye symptoms in South America ranged from 4% to 77.5%, with an average of 39.3%.

Signs of DES brought the prevalence up to 35.2%, with the highest regional rates in Eastern Asia at 42.8% using TFOS DEWS II criteria, indicating a significant global burden of 29.5% with variations across sexes 28.1% in women and 24.9% in men (32). Historically, The estimates of DES prevalence is diverse and ranges from 7.8% to 70.2% (4, 17, 18, 22).

Variations in the prevalence of dry eye symptoms encountered across studies is mostly explained by the difference in the diagnostic criteria indicating the criteria relied on clinical signs versus self-reported symptoms. While self-reported symptoms do provide a measurement of the dry eye status when testing a population, self-reported symptom-based tools (such as the OSDI used in our study) are likely to estimate prevalence lower than the actual prevalence of dry eye symptoms because individuals vary in perception and reporting of discomfort. This suggests we need to be careful about methodology when comparing prevalence across regions.

Comparisons between studies evaluating the prevalence of DES are challenging because of differences in study design, diagnostic criteria, and populations studied (7). Our findings are broadly comparable to those of studies that also relied on symptom-based questionnaires. For example, our values for mild and moderate DES were similar to those reported by Garza-León et al. among university students in Mexico (70.4% overall prevalence, with 19.9% mild, 14.8% moderate, and 35.7% severe) (29). However, the prevalence of severe DES in our sample (26.2%) was higher than that reported by Zhang et al. (23.7%) (34). These discrepancies likely reflect both methodological and contextual differences. Garza-León’s study focused on university students, a group with high screen exposure but relatively young age distribution, while our study included a broader community sample across various age groups. Similarly, Zhang’s study involved adolescents in a different climate zone, where environmental stressors may be less severe than in Jazan.

Environmental and lifestyle factors also contribute to these differences. The hot desert climate of Saudi Arabia, coupled with widespread use of air conditioning in homes and cars, is known to exacerbate tear film evaporation and increase dry eye risk (26, 27). These exposures may explain why our sample demonstrated higher rates of severe DES compared with populations in more temperate climates.

Our results are consistent with other studies showing that female sex is a strong risk factor for DES (35–37). Hormonal influences, differences in tear film composition, and greater prevalence of autoimmune conditions among women may account for this association. Notably, the effect of female sex remained significant in our multivariate model even after adjusting for potential confounders such as age, smoking, screen time, and refractive surgery.

Refractive surgery was associated with higher OSDI scores in unadjusted analyses, consistent with the literature identifying dry eye as the most common postoperative complication (38–40). However, the association did not remain significant after adjustment. This discrepancy may be explained by the absence of data on surgery type and timing in our study, since dry eye symptoms are often most pronounced in the early postoperative period and typically improve over time (39). Future research should incorporate these temporal details to clarify the long-term impact of refractive surgery on DES.

Unlike some earlier studies (7, 18, 29), we found no significant difference in DES prevalence between smokers and non-smokers. This may be partly explained by the relatively small number of smokers in our cohort (n = 166), which limited statistical power, as well as the overall high prevalence of DES in the population. Similarly, although screen exposure is frequently cited as a risk factor, we found no significant association between computer or mobile phone use and DES severity. This aligns with some prior work (29, 41). and suggests that screen exposure alone may not be sufficient to predict dry eye symptoms without considering additional factors such as blinking rate, work environment, or concurrent ocular conditions.

The association between the prevalence of DES and factors such as computer use, mobile phones, and eye surgery is an important area of investigation. The absence of a significant association in this study could be attributed to various factors, including differences in the study populations, methodologies, and sample sizes. The unique characteristics of the Jazan population, such as lifestyle habits, environmental factors, and cultural practices, may have contributed to these differing results. Additionally, variations in the definition and assessment of DES, as well as differences in the tools and questionnaires used, can affect the observed associations. To address this discrepancy, further investigations and robust comparisons with previously published studies are warranted. A comprehensive analysis that considers the specific characteristics of the study population, methodological differences, and potential confounding factors would be valuable for understanding the reasons for the differences in the findings. By conducting such comparisons, the authors can gain insights into the factors contributing to the contrasting results and provide a more comprehensive explanation of the observed associations or lack thereof.

The role of environmental factors in the DES was also explored in this study. The participants were asked to indicate the presence or absence of specific environmental factors related to DES. The results showed that a substantial proportion of the participants reported experiencing certain environmental factors that could contribute to DES. Hot and dry weather was reported by 39.6% of the participants, highlighting the potential impact of climatic conditions on the DES. Air pollution was another significant factor, with 26.9% of participants indicating its presence. Dust and sand particles in the air were reported by 30.2% of the participants, further emphasizing potential irritants in the environment. Exposure to air conditioning or heaters, which can affect humidity levels, was reported by 43.3% of the participants. This finding suggests that artificial heating or cooling systems may contribute to DES symptoms. Lack of humidity, another environmental factor that can influence tear evaporation, was reported by 35.8% of participants. Interestingly, a notable proportion of participants (14.1%) reported experiencing all the aforementioned environmental factors. This indicates the potential cumulative effects of multiple environmental factors on the development and severity of DES. In contrast, a small percentage (8.5%) reported no environmental factors, suggesting that other factors or individual differences might contribute to DES symptoms in these cases.

It is also important to interpret our findings in light of the confidence intervals reported. For example, the association between female sex and DES showed a relatively narrow CI (95% CI: 1.37–2.31), reflecting a precise and consistent effect estimate. In contrast, the association between refractive surgery and DES did not remain statistically significant after adjustment (adjusted OR = 1.53; 95% CI: 0.95–2.47), and the relatively wide CI here indicates uncertainty regarding the true strength of this association. Wide CIs generally result from smaller subgroup sizes, variability in responses, or limited statistical power, and they highlight the need for cautious interpretation. Thus, while our study identifies female sex as a robust risk factor for DES, associations with refractive surgery and other factors should be re-examined in larger studies with sufficient power to narrow CI estimates.

## Limitations

This sampling approach may introduce self-selection bias, as individuals with personal interest in ocular symptoms or access to digital platforms were more likely to participate. This bias may restrict the generalizability of our findings to the entire population of Jazan. Several studies have reported a poor correlation between prevalence rates measured using symptom-based questionnaires and those obtained through objective clinical tests (42, 43). This discrepancy arises because symptom-based tools, such as the Ocular Surface Disease Index (OSDI), rely on self-reported experiences of discomfort, which can be influenced by individual perception and external factors. In contrast, objective clinical tests, such as tear film break-up time (TBUT), Schirmer’s test, and corneal staining, assess physiological changes in the ocular surface but may not always align with a patient’s symptom severity. An additional limitation is the extended data collection period (October 2018 – May 2023), during which seasonal variability in environmental conditions such as temperature, humidity, and allergens may have influenced symptom reporting. Future studies should incorporate seasonal time markers or limit data collection to narrower windows to assess or minimize this variability.

Another limitation is that environmental exposures such as dust, dry air, air conditioning, and humidity were assessed via self-reported, unvalidated survey items, without objective measurement or standardized exposure scales. Participants’ responses may reflect subjective perception rather than actual exposure, and variation in interpretation may affect consistency. Additionally, we did not collect data on contact lens use, which is a well-established risk factor for dry eye symptoms. Future research should incorporate information on lens type, duration of use, and hygiene.

Since no ocular examination was performed in this study to objectively assess clinical signs of DES, our findings reflect only the symptomatic burden of the condition. Given this diagnostic variability, it is widely recommended that future research combine both subjective and objective assessments to ensure a more accurate diagnosis of DES (2). In addition, the Arabic version of the OSDI has been validated (28, 30). We did not evaluate the time required for refractive surgery. Therefore, we could not determine the correlation between the timing of refractive surgery and the development of DES. Other environmental factors, such as contact lens wear (29, 35), and psychological factors, such as stress (44) and autoimmune diseases (44–46), were also not studied. “Another limitation is the extended data collection period (October 2018 – May 2023), during which seasonal variations in climate and allergens in Saudi Arabia may have influenced reported DES symptoms. Dryness and irritation may be more pronounced in hotter months with low humidity or during peak allergy seasons. Since our study did not track seasonal trends, we cannot determine whether symptom prevalence fluctuated throughout the year. Future research should integrate objective clinical tests and explore additional factors influencing DES to enhance diagnostic accuracy and understanding of the condition.

## Conclusion

This study found that dry eye symptoms are highly prevalent in the general population of Jazan Province, Saudi Arabia, with nearly 60% of participants affected. Female sex emerged as a consistent and significant risk factor, while other factors such as screen time and smoking showed no association. These findings highlight the need for targeted screening and public health strategies in high-risk groups within desert environments.

## Data Availability

The original contributions presented in the study are included in the article/supplementary material. Further inquiries can be directed to the corresponding author.

## References

[1] Brewitt H Sistani F . Dry eye disease: the scale of the problem. Surv. Ophthalmol. (2001) 45 Suppl 2:S199–202. doi: 10.1016/S0039-6257(00)00202-2, PMID: 11587143

[2] The definition and classification of dry eye disease: report of the Definition and Classification Subcommittee of the International Dry Eye WorkShop. Ocul. Surf. (2007) 5:75–92. doi: 10.1016/S1542-0124(12)70081-2, PMID: 17508116

[3] Craig JP Nichols KK Akpek EK Caffery B Dua H Joo C-K . TFOS DEWS II Definition and Classification Report. New Zealand (2017) 15(3):276–83. doi: 10.1016/j.jtos.2017.05.008., PMID: 28736335

[4] Lee AJ Lee J Saw S-M Gazzard G Koh D Widjaja D . Prevalence and risk factors associated with dry eye symptoms: a population based study in Indonesia. Br J Ophthalmol. (2002) 86:1347–51. doi: 10.1136/bjo.86.12.1347, PMID: 12446361 PMC1771386

[5] Tong L Waduthantri S Wong TY Saw S-M Wang J Rosman M . Impact of symptomatic dry eye on vision-related daily activities: the Singapore Malay Eye Study. Eye (Lond). (2010) 24:1486–91. doi: 10.1038/eye.2010.67, PMID: 20489740

[6] Pouyeh B Viteri E Feuer W Lee DJ Florez H Fabian JA . Impact of ocular surface symptoms on quality of life in a United States veterans affairs population. Am J Ophthalmol. (2012) 153:1061–66.e3. doi: 10.1016/j.ajo.2011.11.030, PMID: 22330309

[7] Alshamrani AA Almousa AS Almulhim AA Abdullah AA Mohammed AB Abdulrahman AM . Prevalence and risk factors of dry eye symptoms in a Saudi Arabian population. Middle East Afr. J Ophthalmol. (2017) 24:67–73. doi: 10.4103/meajo.MEAJO_281_16, PMID: 28936049 PMC5598305

[8] Bandeen-Roche K Muñoz B Tielsch JM West SK Schein OD . Self-reported assessment of dry eye in a population-based setting. Invest. Ophthalmol Vis Sci. (1997) 38:2469–75., PMID: 9375564

[9] Begley CG Chalmers RL Mitchell LG Nichols KK Caffery B Simpson T . Characterization of ocular surface symptoms from optometric practices in North America. Cornea. (2001) 20:610–8. doi: 10.1097/00003226-200108000-00011, PMID: 11473162

[10] Yu J Asche CV Fairchild CJ . The economic burden of dry eye disease in the United States: A decision tree analysis. Cornea. (2011). doi: 10.1097/ICO.0b013e3181f7f363, PMID: 21045640

[11] Messmer EM . The pathophysiology, diagnosis, and treatment of dry eye disease. Dtsch Arztebl. Int. (2015) 112:71–81. doi: 10.3238/arztebl.2015.0071, PMID: 25686388 PMC4335585

[12] Özcura F Aydin S Helvaci MR . Ocular surface disease index for the diagnosis of dry eye syndrome. Ocul. Immunol Inflamm. (2007). doi: 10.1080/09273940701486803, PMID: 17972223

[13] Alves M Angerami RN Rocha EM . Dry eye disease caused by viral infection: Review. Ribeirão Preto (SP), Brazil (2013). doi: 10.1590/S0004-27492013000200016., PMID: 23828477

[14] Kojima T Dogru M Kawashima M Nakamura S Tsubota K . Advances in the diagnosis and treatment of dry eye. Japan: Keio University School of Medicine (2020). doi: 10.1016/j.preteyeres.2020.100842., PMID: 32004729

[15] Lin P-Y Tsai S-Y Cheng C-Y Liu J-H Chou P Hsu W-M . Prevalence of dry eye among an elderly Chinese population in Taiwan: the Shihpai Eye Study. Ophthalmology. (2003) 110:1096–101. doi: 10.1016/S0161-6420(03)00262-8, PMID: 12799232

[16] Hashemi H Khabazkhoob M Kheirkhah A Emamian MH Mehravaran S Shariati M . Prevalence of dry eye syndrome in an adult population. Clin Experiment. Ophthalmol. (2014) 42:242–8. doi: 10.1111/ceo.12183, PMID: 23927383

[17] Schiffman RM Christianson MD Jacobsen G Hirsch JD Reis BL . Reliability and validity of the ocular surface disease index. Arch Ophthalmol (Chicago Ill. 1960). (2000) 118:615–21., PMID: 10815152 10.1001/archopht.118.5.615

[18] Moss SE Klein R Klein BE . Prevalence of and risk factors for dry eye syndrome. Arch Ophthalmol (Chicago Ill. 1960). (2000) 118:1264–8. doi: 10.1001/archopht.118.9.1264, PMID: 10980773

[19] Schein OD Muñoz B Tielsch JM Bandeen-Roche K West S . Prevalence of dry eye among the elderly. Am J Ophthalmol. (1997) 124:723–8. doi: 10.1016/S0002-9394(14)71688-5, PMID: 9402817

[20] McCarty CA Bansal AK Livingston PM Stanislavsky YL Taylor HR . The epidemiology of dry eye in Melbourne, Australia. Ophthalmology. (1998) 105:1114–9. doi: 10.1016/S0161-6420(98)96016-X, PMID: 9627665

[21] Rege A Kulkarni V Puthran N Khandgave T . A clinical study of subtype-based prevalence of dry eye. J Clin Diagn. Res. (2013) 7:2207–10. doi: 10.7860/JCDR/2013/6089.3472, PMID: 24298477 PMC3843414

[22] Albietz JM . Prevalence of dry eye subtypes in clinical optometry practice. Optom. Vis Sci. (2000) 77:357–63. doi: 10.1097/00006324-200007000-00010, PMID: 10939313

[23] Bukhari A Ajlan R Alsaggaf H . Prevalence of dry eye in the normal population in Jeddah, Saudi Arabia. Orbit. (2009) 28:392–7. doi: 10.3109/01676830903074095, PMID: 19929667

[24] Jie Y Xu L Wu YY Jonas JB . Prevalence of dry eye among adult Chinese in the Beijing Eye Study. Eye (Lond). (2009) 23:688–93. doi: 10.1038/sj.eye.6703101, PMID: 18309341

[25] Tan LL Morgan P Cai ZQ Straughan RA . Prevalence of and risk factors for symptomatic dry eye disease in Singapore. Clin Exp Optom. (2015) 98:45–53. doi: 10.1111/cxo.12210, PMID: 25269444

[26] Wolkoff P Nøjgaard JK Franck C Skov P . The modern office environment desiccates the eyes? Indoor Air. (2006) 16:258–65. doi: 10.1111/j.1600-0668.2006.00429.x, PMID: 16842606

[27] Wolkoff P Nøjgaard JK Troiano P Piccoli B . Eye complaints in the office environment: precorneal tear film integrity influenced by eye blinking efficiency. Occup. Environ Med. (2005) 62:4–12. doi: 10.1136/oem.2004.016030, PMID: 15613602 PMC1740860

[28] Bakkar MM El-Sharif AK Al Qadire M . Validation of the Arabic version of the ocular surface disease index questionnaire. Int J Ophthalmol. (2021). doi: 10.18240/ijo.2021.10.18, PMID: 34667738 PMC8481982

[29] Garza-León M Valencia-Garza M Martínez-Leal B Villarreal-Peña P Marcos-Abdala HG Cortéz-Guajardo AL . Prevalence of ocular surface disease symptoms and risk factors in group of university students in Monterrey, Mexico. J Ophthalmic Inflamm Infect. (2016) 6:44. doi: 10.1186/s12348-016-0114-z, PMID: 27864795 PMC5116015

[30] Aljarousha M Badarudin NE Che Azemin MZ Aljeesh Y Amer A Abdul Rahim MAS . The validity and reliability of the Arabic version of the ocular surface disease index (OSDI) questionnaire in a sample of the Gazan population: a study from Palestine. Int Ophthalmol. (2023). doi: 10.1007/s10792-022-02528-7, PMID: 36156181

[31] Chen H McCann P Lien T Xiao M Abraham AG Gregory DG . Prevalence of dry eye and Meibomian gland dysfunction in Central and South America: a systematic review and meta-analysis. BMC Ophthalmol. (2024) 24:1–23. doi: 10.1186/s12886-023-03249-w, PMID: 38297204 PMC10829227

[32] Papas EB . The global prevalence of dry eye disease: A Bayesian view. Ophthalmic Physiol Opt. (2021) 41:1254–66. doi: 10.1111/opo.12888, PMID: 34545606

[33] Loaiza-Guevara V Salazar-Santoliva C Villota-Arevalo AJ Acosta-Villas ME Coral-Gaón B-L Afanador JE . Understanding the dry eye disease-related symptoms in South America: prevalence and associated factors-A systematic review. J Clin Med. (2024) 13. doi: 10.3390/jcm13206060, PMID: 39458010 PMC11508735

[34] Zhang Y Chen H Wu X . Prevalence and risk factors associated with dry eye syndrome among senior high school students in a county of Shandong Province, China. Ophthalmic Epidemiol. (2012) 19:226–30. doi: 10.3109/09286586.2012.670742, PMID: 22650150

[35] Uchino M Dogru M Uchino Y Fukagawa K Shimmuraa S Takebayashic T . Japan Ministry of Health study on prevalence of dry eye disease among Japanese high school students. Am J Ophthalmol. 146:925–9.e2:2008. doi: 10.1016/j.ajo.2008.06.030, PMID: 18723141

[36] Uchino M Schaumberg DA Dogru M Uchino Y Fukagawa K Shimmura S . Prevalence of dry eye disease among Japanese visual display terminal users. Ophthalmology. (2008) 115:1982–8. doi: 10.1016/j.ophtha.2008.06.022, PMID: 18708259

[37] Jamaliah R Fathilah J . Prevalence of dry eye in University Malaya Medical Centre. Med J Malaysia. (2002) 57:390–7., PMID: 12733162

[38] Xu Y Yang Y . Dry eye after small incision lenticule extraction and LASIK for myopia. J Refract. Surg. (2014) 30:186–90. doi: 10.3928/1081597X-20140219-02, PMID: 24763723

[39] Patel SV McLaren JW Kittleson KM Bourne WM . Subbasal nerve density and corneal sensitivity after laser in *situ* keratomileusis: femtosecond laser vs mechanical microkeratome. Arch Ophthalmol (Chicago Ill. 1960). (2010) 128:1413–9. doi: 10.1001/archophthalmol.2010.253, PMID: 21060042 PMC3911824

[40] Chao C Golebiowski B Stapleton F . The role of corneal innervation in LASIK-induced neuropathic dry eye. Ocul. Surf. (2014) 12:32–45. doi: 10.1016/j.jtos.2013.09.001, PMID: 24439045

[41] Unlü C Güney E Akçay Bİ.S Akçalı G Erdoğan G Bayramlar H . Comparison of ocular-surface disease index questionnaire, tearfilm break-up time, and Schirmer tests for the evaluation of the tearfilm in computer users with and without dry-eye symptomatology. Clin Ophthalmol. (2012) 6:1303–6. doi: 10.2147/OPTH.S33588, PMID: 22927744 PMC3422136

[42] Hua R Yao K Hu Y Chen L . Discrepancy between subjectively reported symptoms and objectively measured clinical findings in dry eye: a population based analysis. BMJ Open. (2014) 4:e005296. doi: 10.1136/bmjopen-2014-005296, PMID: 25168038 PMC4156796

[43] Mizuno Y Yamada M Miyake Y Dry Eye Survey Group of the National Hospital Organization of Japan . Association between clinical diagnostic tests and health-related quality of life surveys in patients with dry eye syndrome. Jpn J Ophthalmol. (2010) 54:259–65. doi: 10.1007/s10384-010-0812-2, PMID: 20700790

[44] Ahn JM Lee SH Taek Rim TH Park RJ Yange HS Kim TI . Prevalence of and risk factors associated with dry eye: the Korea National Health and Nutrition Examination Survey 2010-2011. Am J Ophthalmol. (2014) 158:1205–1214.e7. doi: 10.1016/j.ajo.2014.08.021, PMID: 25149910

[45] Her Y Lim JW Han SH . Dry eye and tear film functions in patients with psoriasis. Jpn J Ophthalmol. (2013) 57:341–6. doi: 10.1007/s10384-012-0226-4, PMID: 23525680

[46] Lee S-Y Petznick A Tong L . Associations of systemic diseases, smoking and contact lens wear with severity of dry eye. Ophthalmic Physiol Opt. (2012) 32:518–26. doi: 10.1111/j.1475-1313.2012.00931.x, PMID: 22958181

